# Reanalyzing the Maia and McClelland (2004) Empirical Data: How Do Participants Really Behave in the Iowa Gambling Task?

**DOI:** 10.3389/fpsyt.2022.788456

**Published:** 2022-04-08

**Authors:** Yao-Chu Chiu, Jong-Tsun Huang, We-Kang Lee, Ching-Jen Lin, Ching-Hung Lin

**Affiliations:** ^1^Department of Psychology, Soochow University, Taipei, Taiwan; ^2^Graduate Institute of Biomedical Sciences, China Medical University, Taichung, Taiwan; ^3^Sleep Center, Shin Kong Wu Ho-Su Memorial Hospital, Taipei, Taiwan; ^4^Department of Psychology, Kaohsiung Medical University, Kaohsiung, Taiwan; ^5^Research Center for Nonlinear Analysis and Optimization, Kaohsiung Medical University, Kaohsiung, Taiwan

**Keywords:** implicit emotion, explicit knowledge, gain–loss frequency, Iowa Gambling Task, myopic, foresight, somatic marker hypothesis

## Abstract

**Background:**

Since 2007, the Iowa Gambling Task (IGT) has been a standardized clinical assessment tool for assessing decision behavior in 13 psychiatric/neurological conditions. After the publication of Maia and McClelland's (1) article, there were two responses in 2005 from Bechara et al. and Maia and McClelland, respectively, discussing whether implicit emotion or explicit knowledge influences the development of foresighted decision strategies under uncertain circumstances (e.g., as simulated in the IGT).

**Methods and Results:**

We reanalyze and verify the data obtained by Maia and McClelland (1) in their study “What participants really know in the Iowa Gambling Task” and find that decision-makers were lured into shortsighted decisions by the prospect of immediate gains and losses.

**Conclusion:**

Although the findings of this reanalysis cannot support any arguments concerning the effect of either implicit emotion or explicit knowledge, we find evidence that, based on the gain–loss frequency in the IGT, participants behave myopically. This is consistent with most IGT-related articles (58 out of 86) in Lee et al.'s (2) cross-cultural review. Alternatively, under uncertain circumstances, there is probably no such thing as foresighted decision strategy irrespective of the proposed mechanisms of implicit emotion or explicit knowledge.

## Introduction

Emotion has long been perceived as an uncontrollable horse (3) (e.g., in Plato's *Phaedrus*), the opposite of rationality. The Eighteenth-century philosopher Hume proposed the view that rationality is subservient to emotion (4). While a variety of arguments regarding the tension between emotion and rationality have been made in philosophy, literature (especially Shakespeare's works) (5), and psychology (e.g., Darwin and James) (6, 7), few studies [e.g., (8)] used an empirical approach to explore the influence of emotion and rationality on decision-making. In light of this omission, Damasio (9) and other researchers (10, 11) sought to address this gap by proposing the **somatic marker hypothesis** (SMH), which holds that emotion is not subservient to rationality and, instead, has a positive effect on how rationality operates.

Bechara et al. (10) designed the **Iowa Gambling Task** (IGT) to verify the SMH (9) formulated by their University of Iowa research team, thereby creating an important theory and a tool for studying issues relating to emotion and decision-making. However, its conceptualization has also attracted a series of critiques (12), of which Maia and McClelland's (1) is one of the most prominent. Bechara et al. (10, 11) proposed that **implicit emotion** would help a healthy decision-maker in an IGT experiment to develop foresighted decision strategies allowing gains to be made. However, Maia and McClelland (1) argued that a participant could develop such strategies without having to tap into their emotions, as they would have already acquired **explicit knowledge** regarding gains during the earlier phases of the experiment. Among researchers, this debate has become the classic framework within which to examine the SMH, first developed by Damasio et al. (9–11).

In 2005, a research focus article (13) and a corresponding research focus response (14) were published debating whether implicit emotion or explicit knowledge dominate prescient decision behavior (e.g., pursuing the choice of a positive final outcome) in conditions of uncertainty. Following up on Maia and McClelland's (1) earlier critique, Bechara et al. (13), in their article, sought to address the issues that had been raised with respect to their original work (10, 11). Bechara et al. (13) considered that Maia and McClelland's (1) behavioral illustration was consistent with many economists' findings that decision-makers could be guided to the deviate choice depending on the guidance of prior knowledge. Therefore, they considered Maia and McClelland's (1) finding not to be harmful to the SMH. The SMH demonstrated that emotion plays a key role in decision-making under the unconscious and conscious process and provided the possible physiological evidence to illustrate this (13).

However, Maia and McClelland (14) countered that Bechara et al.'s latest account (13) elicited “many questions but no answers.” Maia and McClelland (14) emphasized that their research finding (1) did not aim to show that the SMH was inaccurate but demonstrated that there are relatively simple alternative explanations regarding healthy decision-makers' behavior in the IGT. Healthy decision-makers developed explicit knowledge of the decks of cards in the very early stage that Bechara et al. (10, 11) observed. In other words, the SMH was not necessary to explain the decision behavior in the IGT. Therefore the IGT was redundant (14). The two research teams had not reached a consensus, and a significant issue remained unresolved.

In the present study, we discuss the unresolved issue in detail, and we reanalyze Maia and McClelland's (1) original data. We found that the data did not support the basic viewpoints of Maia and McClelland (14) or Bechara et al. (13) in terms of long-term outcomes. This issue initially arose in Maia and McClelland's (1) arguments against Bechara et al.'s IGT studies (10, 11). Our study reanalyzed the 2004 raw data (15) generated by Maia and McClelland (1) and found that the participants in that experiment preferred frequent and immediate gains, leading them to adopt myopic and ultimately loss-making strategies. This finding indicates that the implicit emotion and explicit knowledge components proposed by Bechara et al. (10, 11) and by Maia and McClelland (1), respectively, cannot provide a clear explanation concerning the participants' adoption of myopic decision strategies. Therefore, through a reanalysis of Maia and McClelland's (1) raw data, the present study sought to reinterpret these data from the perspective of **gain–loss frequency**.

### Participants' Knowledge in the Iowa Gambling Task

Four decks of cards are used for the IGT (Decks A, B, C, and D; see Table 1), and each deck has a different gain–loss structure. With each block consisting of 10 trials, every time a card is drawn from Decks A or B, it is possible to win $100 or lose money. The number of times a participant can lose money is not fixed. For the first 10 trials of Deck A, the third, fifth, seventh, ninth, and tenth cards could lead to a loss of $150, $300, $200, $250, and $350, respectively. When drawing cards from Deck B, the ninth card could lead to a loss of $1,250. Every time a card is drawn from Deck C or D, it is possible to win $50 or lose money. For the first 10 trials of Deck C, the third, fifth, seventh, ninth, and tenth cards could lead to a loss of $50. For Deck D, the tenth card could lead to a loss of $250.

**Table 1:** IGT gain–loss structure.

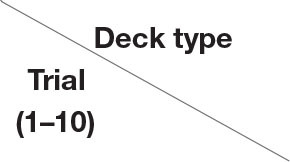	**A**		**B**		**C**		**D**	
	**Bad deck**		**Bad deck**		**Good deck**		**Good deck**	
1	100		100		50		50	
2	100		100		50		50	
3	100	**−150**	100		50	**−50**	50	
4	100		100		50		50	
5	100	**−300**	100		50	**−50**	50	
6	100		100		50		50	
7	100	**−200**	100		50	**−50**	50	
8	100		100		50		50	
9	100	**−250**	100	**−1,250**	50	**−50**	50	
10	100	**−350**	100		50	**−50**	50	**−250**
Final outcome (expected value)	**−250**		**−250**		**250**		**250**	
Number of gains/losses	**10 wins**		**10 wins**		**10 wins**		**10 wins**	
	**5 losses**		**1 loss**		**5 losses**		**1 loss**	
Net gain-loss	**5 wins**		**9 wins**		**5 wins**		**9 wins**	
	**5 losses**		**1 loss**		**5 ties**		**1 loss**	

***Trials (1–10)** = the gain–loss state for each card in the four decks (Decks A, B, C, and D) under the original IGT's 10-trial gain–loss structure, participants would gain and loss money at the same time each trial. For example, the ninth trial of Deck B could lead to a gain of $100 and a loss of $1,250. However, when the IGT was performed using a 20-trial gain–loss structure, there were slight changes in the order of the cards and the corresponding amount of gains or losses (**Trials 11–20**). For example, in the 11th to the 20th trials for Deck B, the 14th trial could lead to a gain of $100 and a loss of $1,250. **Final outcome (expected value)** = tallied results for gains and losses over 10 trials. **Number of gains/losses** = total number of wins and losses over 10 trials. **Net gain–loss** = tallied results obtained after adding the amount gained and lost for each card over 10 trials. Source: Table content is based on (10, 16)*.

Assuming that a block consisting of 10 trials is used as the standard for calculations, a participant who continues to choose Decks A or B for 10 trials will suffer a loss of $250 (final outcome/expected value), while a participant who chooses Decks C or D for 10 trials will win $250 (final outcome). Based on the final outcomes, Decks A and B are considered “bad” or disadvantageous decks. In terms of numbers of gains and/or losses and net gain–loss, Deck A generates five wins and five losses. Deck B contains many cards that lead to gains (i.e., nine wins and one loss). However, the card that could lead to a loss results in a considerable loss (i.e., $1,250). Decks C and D are considered “good” or advantageous decks, with Deck C having a net gain–loss of five wins and five ties, and Deck D containing many cards that lead to gains (nine wins and one loss). Although Deck D also contains a card that could lead to a significant loss (i.e., $250), the amount involved is much lower than the Deck B loss (i.e., $1,250). The net gain–loss values for Decks B, C, and D indicate that these decks offer frequent gains and infrequent losses (see Table 1). If a participant favors Deck B—a bad deck—during an IGT, this preference is referred to as a **prominent Deck B phenomenon** (PDB phenomenon) (17, 18).

### Bechara et al.'s Research Results

Damasio et al. and Bechara et al. (9, 10) believed that the high degree of uncertainty in our world makes it difficult to rely solely on logical reasoning to manage ever-changing and complex situations. They also proposed that, in an appropriate situation, implicit emotion could help us make decisions. This view was tested by Bechara et al. (11) in their IGT experiment, which tracked the deck selection behavior of patients with **ventromedial prefrontal cortex** (vmPFC) lesions and of non-patients (“healthy” participants). They also measured participants' **galvanic skin responses** (GSR) during the deck selection process.

During the IGT experiment, a participant could choose to draw cards from any of the four decks, and immediate gain–loss feedback was provided when a card was drawn (see Table 1). The experimental process comprised 100 trials. However, the participants did not know the number of trials involved. At the start of the experiment, the two groups of participants showed a preference for the bad decks (A and B), which offered higher gains and losses but eventually led to losses. However, the healthy participants gradually gravitated toward the good decks (C and D), which offered lower gains and losses but ultimately led to gains. In contrast, the vmPFC patients continued to choose the bad decks. During the deck selection process, the healthy participants experienced changes in their GSR levels, while the vmPFC patients' GSR levels remained low. These findings indicated that, when faced with a bad deck, healthy participants were guided by their implicit emotion toward making advantageous decisions. In contrast, the vmPFC patients were unable to resist the bad decks due to their lack of implicit emotion, eventually causing them to suffer losses.

Bechara et al.'s (11) study demonstrated that, for normal participants, their gain–loss experience was recorded by their implicit emotion systems each time they drew a card. Between the early and late stages of the game, these participants gradually acquired knowledge about the pros and cons of each deck, and as a result, even though they were initially unsure about the quality of each deck, their somatic markers gradually guided them away from the disadvantageous decks and to the advantageous ones. Bechara et al. (11) stressed the importance of implicit emotion in rational decision-making as follows:

The results suggest that, in normal individuals, nonconscious biases guide behavior before conscious knowledge does. Without the help of such biases, overt knowledge may be insufficient to ensure advantageous behavior.

Bechara et al. (11) (p. 1293)

### Maia and McClelland's Research Results

Concerning the IGT experimental structure, Maia and McClelland (1) conceived that normal participants would not have to rely on their implicit emotion when engaging in decision-making, because a decision-maker could acquire knowledge of the game during an early stage of the experiment. They argued that Bechara et al. (11) had been unable to observe participants' understanding of game knowledge because the questionnaire they used to measure the game knowledge of decision-makers was not sufficiently sensitive.

During the IGT experiment, to measure the level of game knowledge they had acquired, Bechara et al. asked the participants about their knowledge and feelings. Maia and McClelland (1) posited that these open-ended questions were too vague and made it difficult for participants to provide proper answers regarding their game knowledge. For this reason, Maia and McClelland (1) designed a new questionnaire with items that allowed participants to evaluate the quality of each deck, to provide the reasons behind their evaluations, and to indicate whether they understood the average net result, average win and loss result, and the number of losses expected over 10 trials for each deck. The items in this revised questionnaire enabled Maia and McClelland (1) to measure the participants' understanding of game knowledge directly.

Maia and McClelland's experiment (1) involved two groups. The first group participated in the replication experiment, in which the approach of Bechara et al. (11) (IGT experiment and two-item questionnaire) was utilized (Supplementary Figure 1). The second group participated in line with Maia and McClelland's approach (1) (IGT experiment and newly designed questionnaire). Each group had 20 participants, and no vmPFC patients or GSR measurements were included in the two approaches. The participants each underwent 100 trials, and their deck selection preferences for the four decks were recorded. Using Maia and McClelland's methodology, the corresponding questionnaire was used to measure participants' understanding of game knowledge. The experimental results revealed the following: First, there was a consistent relationship between participants' deck selection preferences and their game knowledge for those participants using Maia and McClelland's (1) approach [see Figure 2 in (11); Supplementary Figure 2]. Second, participants following the procedures of Bechara et al. (11) and Maia and McClelland (1) drew from the good decks 58.6 and 63.55 times, respectively (see Table 2). Third, no differences between the two groups were identified concerning the frequency with which the good decks were selected (see p. 2 of the Online Supplemental Information on Maia and McClelland's research). Although the questionnaire used in (1) contained many in-depth items, this did not influence the participants' deck selection preferences. The study by Maia and McClelland (1) successfully replicated the results obtained by Bechara et al. (11), whereby the preference for good decks among the healthy participants was the same:

This analysis shows that, by using the methods of Bechara et al. … we replicated their statistically significant results; specifically, participants behaved advantageously when they were classified according to the criteria of Bechara et al. as being in either the hunch or conceptual periods (our Levels 1 and 2, respectively).

Maia and McClelland (1) (p. 16077)

**Table 2:** Deck selection frequency: Bechara et al. (11) vs. Maia and McClelland (1).

**IGT decks**	**Experimental approach**	
	**Bechara et al. (11)**	**Maia and McClelland (1)**
Deck A	14.40	14.50
Deck B	27.00	21.95
Bad decks (Deck A+B)	*41.40*	*36.45*
Deck C	30.65	30.95
Deck D	27.95	32.60
Good decks (Deck C+D)	*58.60*	*63.55*

Maia and McClelland's (1) questionnaire (Supplementary Figure 2) also generated more precise measurements relative to Bechara et al.'s (11) questionnaire. This key study indicated that, during the early period of the experiment, participants already possessed explicit knowledge regarding the good and bad decks, and that this was reflected in their subsequent deck selection preferences. Accordingly, Maia and McClelland (1) inferred that implicit emotion is not required for the decision-making process—thereby clearly contradicting the SMH position. The respective studies adopted different positions concerning the role of implicit emotion and explicit knowledge in the formulation of foresighted strategies under uncertain circumstances.

Table 2 shows our analysis of participants' deck selection behavior under the two approaches [i.e., as reported in (11) and (1)]. The data indicate that cards were drawn from Deck B 27.00 and 21.95 times, respectively. In the two approaches, participants demonstrated a stronger preference for the bad Deck B than for the bad Deck A. However, based on the SMH, healthy participants should be (a) avoiding Decks A and B, and (b) showing similar preference levels for Decks A and B, as the results in the original IGT study showed (10).

Maia and McClelland's results (1) did not indicate the frequency with which the four decks were selected. Neither did they provide a direct description or verification of the preference for Deck B, as observed for the two approaches. However, our study reanalyzed Maia and McClelland's (1) data and created Table 2, which shows the number of times the four decks were selected and confirms the participants' preference for Deck B.

## Methods

### A Reanalysis of Maia and McClelland's Research

Our study reanalyzed Maia and McClelland's data (1), specifically the number of times the participants selected each of the four decks. The data were first analyzed based on Maia and McClelland's open data (1) published in Steingroever et al. (15), but the participants' approaches were not clearly identified in this data. Therefore, to obtain the original data, Maia and McClelland (1) were contacted directly. The data extracted from (15) were then rechecked and rearranged in line with the original data (1). Supplementary Table 1 shows the data rearranged for easy comparison and statistical testing.

## Results

Two-way ANOVA (conditions ^*^ decks) performed in our study indicated that there were no interaction effects between conditions (Maia vs. Bechara) and decks (A, B, C, D) (the analysis showed that the distribution did not meet the spherical hypothesis, and the Greenhouse–Geisser correction was adopted). However, the main effect of decks was shown to be significant [*F*_(1.836, 69.766)_ = 9.343, *p* < 0.001, $\eta_{p}^{2}$ = 0.197].

**Figure 1:**
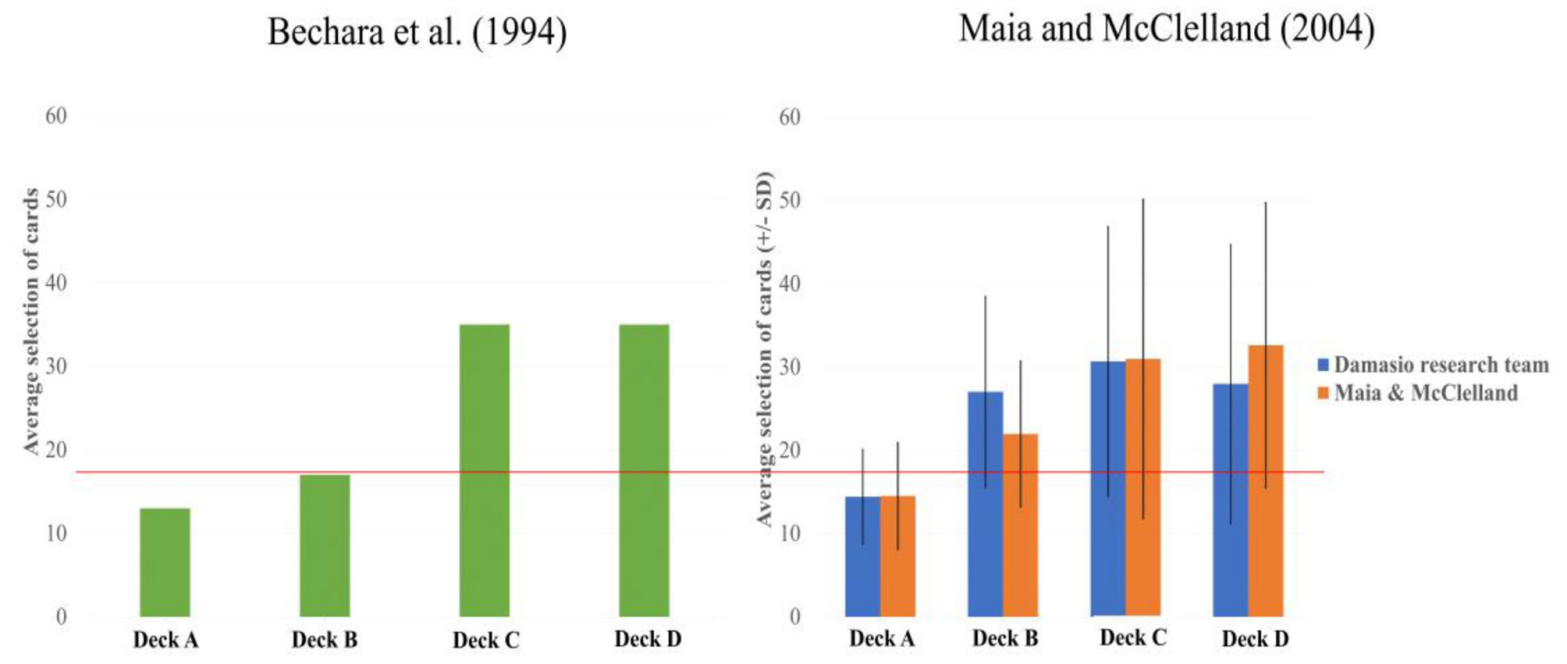
The average deck selection frequency for the four IGT decks. Left-hand chart: data from the original experiment, as generated from Bechara et al. (10). Right-hand chart: the orange bars represent the data from Maia and McClelland (1), while the blue bars represent the results obtained by Maia and McClelland when they replicated the Bechara et al. (11) approach. This chart was generated from Maia and McClelland's (1) original data. The right-hand chart presents the average deck selection frequency in Bechara et al. (11) and the study of Maia and McClelland (1), taking their different methodologies (see also Supplementary Figures 1, 2) into account. The analysis shows that, following Bechara et al.'s (11) procedures, there was no difference in terms of participants' preferences for Decks B, C, and D. However, participants showed a lower preference for Deck B than for Deck D, indicating that Maia and McClelland's questionnaire had influenced participants by alerting them to the negative properties of Deck B, thereby reducing the frequency with which Deck B was selected. It should be noted that participants in both studies selected Deck B more often than Deck A, a result that counters the original hypothesis proposed by Damasio and by Bechara et al. (9–11), as well as the view held by Maia and McClelland that participants possessed explicit knowledge relating to gains. The color bars represent the mean number of card selections in each deck, and the error bars mark the 1 positive/negative standard deviation from the mean selection number of each deck. Due to the limited number of participants in Maia and McClelland's study, the error bars are only for presentation purposes and not for data correction.

We conducted further *post hoc* analysis using a repeated measures method (one-way ANOVA) to analyze participants' deck selection preferences under each of the two approaches (19–21). Significant differences were identified in the results for the Maia and McClelland's study (1) [*F*_(1.640, 31.162)_ = 5.483, *p* < 0.05, $\eta_{p}^{2}$ = 0.224] and for the Bechara et al.'s study (11) [*F*_(2.003, 38.057)_ = 4.448, *p* < 0.05, $\eta_{p}^{2}$ = 0.190], concerning the number of times each of the four decks was selected (see Figure 1) (both distributions did not meet the spherical hypothesis, and the Greenhouse–Geisser correction was adopted). An LSD *post hoc* analysis indicated that, for the Maia and McClelland's study (1), the number of times Deck A was selected was significantly lower compared to the number of times Decks B (*p* < 0.001), C (*p* < 0.01), and D (*p* = 0.001) was selected. There was nonsignificant difference between Decks B and C, and between Decks C and D, in terms of the number of times they were selected, although the number of times Deck B was selected was significantly lower compared to the number of times Deck D (*p* < 0.05) was selected. For the Bechara et al. (11) study, the LSD *post hoc* analysis indicated that the number of times Deck A was selected was significantly lower compared to the number of times Decks B (*p* < 0.001), C (*p* = 0.001), and D (*p* < 0.01) was selected. Between Deck B, C, and D, the mean number of card selection was a lack of statistical significance.

In order to compare further the effect of conditions [Maia and McClelland (1) vs. Bechara et al. (11)] on the number of times that each deck (Decks A, B, C, and D) was selected and the good deck (Decks C and D) indicators, we performed the independent samples *t*-test. The results indicated that, regarding the number of times each deck was selected and the good deck indicators, there were no statistically significant differences between the approaches of (11) and (1).

## Discussion

This reanalysis of Maia and McClelland's (1) data revealed two new phenomena. First, the questionnaire designed by Maia and McClelland allowed participants to focus better on the final outcome (expected value), with the bad Deck B being selected less often (21.95) than the good Deck D (32.60) (see Table 2), whereas, in the Bechara et al. (11) data, no differences were found between the bad Deck B and Decks C and D, thereby challenging the contention of Maia and McClelland (1) that the use of the questionnaire would only have a slight impact on participants' deck selection preferences. In addition, a separate study indicated that the Maia and McClelland questionnaire (1) does influence participants' decision-making (22, 23). Second, under the methodologies of (11) and (1), participants not only demonstrated a preference for Decks C and D (which offered frequent gains) and avoided Deck A (which led to frequent losses) but also exhibited a preference for Deck B (which offered frequent gains but ultimately led to losses); in other words, the PDB phenomenon. In addition, the Yen (23) study adopted the paradigm of the Maia and McClelland (1) experiment and also observed that the number of times Deck B was selected was more often than Deck A.

The Maia and McClelland study (1) did not generate results that were consistent with the views proposed in the original research conducted by Damasio et al. (10, 11) (see the chart on the left in Figure 1). All the participants, under the two approaches, demonstrated a preference for the bad Deck B. This is counter to the proposition that healthy participants will prefer the good decks and adopt a foresighted strategy. The consistent preference of participants for Decks B, C, and D and their avoidance of Deck A indicates that the gain–loss frequency factor was better able to guide participants' decision-making behavior—more than the implicit emotion and explicit game knowledge concepts proposed in Bechara et al. (11) and Maia and McClelland (1), respectively.

While the results from our reanalysis of Maia and McClelland's (1) data do not correspond to the argument proposed in that study, our finding that gain–loss frequency influenced the IGT performance of participants is not novel. It matches the findings of several prior studies (18, 24–26) that questioned the foresighted strategy concept proposed by Damasio and by Bechara et al. (9–11). The results obtained from our reanalysis of Maia and McClelland's (1) data are consistent with those from studies that revealed a preference for Deck B (2).

### Random Deck Selection

Our analysis of the additional research data released to us by Maia and McClelland indicated that two participants, participants no. 36 and no. 41 [see p. 9–12, 15 of the Online Supplemental Information (1)], exhibited inconsistencies in their preference-related decision behavior and game knowledge. Maia and McClelland (1) defined this alternative preference-related behavior as **random deck selection**. For example, during the 30th trial, participant no. 41 experienced a second major loss with Deck B, after which they appeared to select the decks in a random manner. A further examination of participant no. 41's deck selection behavior revealed that Decks A, B, C, and D were selected 22, 32, 17, and 29 times [analysis based on data generated by Maia and McClelland (1), Supplementary Figure 7 on p. 9 of the Online Supplemental Information], indicating that participant 41 favored the bad Deck B and the good Deck D, both of which offered frequent gains.

According to the Online Supplemental Information containing Maia and McClelland's (1) research [(17), p. 9–12], participant no. 36 selected Deck B 11 times during the first 20 trials, resulting in a loss and a negative net final outcome. Although Deck B accounted for the biggest losses suffered by this participant, the description they provided regarding this choice revealed an understanding of Deck B as a deck that could lead to sudden and substantial losses, but also to substantial gains. Therefore, participant no. 36 determined that Deck B was a good deck and not a bad deck regarding the potential overall gains and losses (final outcome/expected value). In participant no. 36's oral report, they made the following observations regarding this matter:

Because it seems good, because I won a lot of money in the beginning, and then all of a sudden, I lost, but it seemed like you could win a lot of money.

[Maia and McClelland (1); Online Supplemental Information, p. 13]

Maia and McClelland (1) concluded that participants 36 and 41 had adopted a random selection strategy. Their behavior contradicted the researchers' hypothesis that participants would acquire knowledge regarding the good and bad decks during the early stage of the experiment. However, the two participants' deck selection behavior regarding Decks B and D also reflected their preference for frequent and immediate gains, matching the researchers' observations regarding gain–loss frequency (2). When we investigated the number of times Deck B was selected by the participants who followed Bechara et al.'s procedures (11) (27 times) and those of Maia and McClelland (1) (21.95 times), it is clear that Deck B was selected significantly more often than Deck A, and that both decks accounted for approximately a quarter (48.95/200) of the 200 trials—or, roughly, the average selection frequency for the four decks. These findings suggest that the participants in the study (1) did not strongly perceive Deck B to be a bad deck (see Table 2 and the chart on the right in Figure 1). The various analyses—including those regarding participants 36 and 41, the analysis of the participants' preference for Deck B in both (11) and (1), and the analysis of the differences between the participants' preference for Decks A and B under both methodologies—that were carried out all indicated that the participants' deck selection strategy was not random. In addition, the gain–loss frequency had influenced their adoption of a **myopic decision strategy** when engaging in deck selection. This observation regarding gain–loss frequency contradicts the positions advanced in Bechara et al.'s (13) article and Maia and McClelland's response (14) concerning implicit emotion and explicit knowledge.

### The Myopic Decision Strategy and Its Significance

Maia and McClelland (1) concluded that their questionnaire could effectively measure game-related explicit knowledge. Based on the assumption that participants could acquire gain-related knowledge through their deck selection experience during the early stage of the experiment, the researchers theorized that participants would not need to rely on their implicit emotions to develop a strategy characterized by a preference for good decks. Our study reanalyzed Maia and McClelland's (1) experimental data (1) and discovered that participants in that study had demonstrated a preference for bad Deck B over bad Deck A (i.e., the PDB phenomenon), suggesting that Maia and McClelland had not replicated the results from the original study (11), but had obtained results that contradicted prior views relating to implicit emotion and explicit knowledge. Although Maia and McClelland classified participant no. 41's decision-making behavior as a random strategy, it seems that this behavior was guided by gain–loss frequency and was not random. Similarly, participant no. 36's preference for Deck B during the early stage of the experiment (as indicated in the oral report) also indicates the influence of gain–loss frequency (2). Moreover, Maia and McClelland (1) believed that their questionnaire did not influence the participants' IGT performance. This is because Maia and McClelland analyzed the good decks (C and D) as a single entity. They did not examine participants' preferences for each of the four decks. However, our study considered the number of times each of the four decks was selected. We found that participants following Maia and McClelland's methodology selected the bad Deck B less often than those adhering to the approach identified in the study by Bechara et al. (11) (Table 2), a finding that suggests that the questionnaire influenced the experiment.

In proposing the concept of explicit knowledge and questioning the necessity of implicit emotion, Maia and McClelland's research (1) became a classic study used to examine the SMH. However, our analysis of Maia and McClelland's original data revealed that the participants had adopted a myopic decision strategy, thereby contradicting the inferences regarding explicit knowledge and implicit emotion. We posit that, compared to explicit knowledge and implicit emotion, gain–loss frequency should enable a more reasonable explanation. Moreover, it is consistent with the PDB phenomenon that several recent IGT-related studies have proposed (2, 17, 18, 24–26).

### Cross-Cultural PDB Phenomenon and Clinical Implications

Lee et al. (2) considered 86 IGT-related studies that presented their data in the four-deck format, which allowed the mean number of each deck to be clearly compared and analyzed. This review showed that 58 out of 86 studies indicated the presence of the PDB phenomenon.

The above research shows that the performance of the control group is the basic reference point for comparison with clinical cases. Therefore, in our review, we revisited 41 out of these 86 studies, including the experimental groups of clinical cases diagnosed by the DSM or ICD system and the clinical cases of individuals with brain injuries (41/86 articles, 47.67%).

In the 41 IGT clinical studies, for the control groups, there were 23 studies where the mean number of Deck B selections was greater than 25, indicating that the PDB phenomenon was present (23/41, 56.1%). However, 28 studies had a significant net score difference between the experimental and control groups, indicating that, based on this survey, the net score might still be an effective differential index in most IGT clinical studies (28/41, 68.29%). Notably, 12 studies simultaneously revealed the PDB phenomenon and a significant net score between the experimental and control groups (12/41, 29.27%). This observation indicated that about 1/3 of studies revealed that two contradictory phenomena co-exist in the same studies. In addition, in 37 studies, the mean number for Deck B selection was larger than 20 (37/41, 90.24%) for the control group. An additional survey indicated that five studies identified that the mean number for Deck B selection was significantly larger than that for Deck A (5/41, 12.2%). Four studies indicated that the mean number for Deck B selection was significantly larger than for Deck A, and the mean number for Deck B selection was significantly larger than 20 (4/41, 9.76%). Moreover, only two studies demonstrated that Deck B selection was significantly larger than Deck A selection and that the net score was significant (2/41, 4.88%). Only one study (27) completely matched the results of Maia and McClelland (1) (B > 20, B > A, significant net score in control group).

In short, the present survey found that bad Decks B and A are not consistently avoided by the control group, as assumed in the original literature (1, 10, 11). This may be the main factor indicating that some clinical literature cannot distinguish between the behavioral performance of the experimental and the control groups based on the net score index (11/41, 26.83%). Nevertheless, the net score can distinguish effectively between the experimental and control groups in over 50% of studies (28/41, 68.29%). Therefore, the net score is not completely irrelevant. The PDB phenomenon that is identified is only inconsistent with the original assumption that the control group is assumed to choose more cards with high long-term outcomes (10). Consequently, this means that gain–loss frequency might be the most dominant guiding factor in decision behavior under uncertainty (2) and that the final outcome/expected value might be a secondary factor. Furthermore, an increasing number of IGT clinical studies compared the selection strategies of neurological/psychiatric patients and control groups found that the control group participants chose Deck B significantly more often than Deck A (24, 27–31). The number of clinical cases exhibiting the PDB phenomenon during IGT has yet to be established. There is a need for a global survey of experimental groups in IGT clinical studies.

### Back to Plato's Chariot Allegory

Even though the emergence of SMH has led to a stronger focus on emotion-related topics, an increasing number of studies that contradict the hypothesis seem to be returning to the supposition that the role of emotion in decision-making is, as in Plato's allegory, a “difficult-to-control chariot.” Explicitly, it is difficult to rein in emotions through the application of rationality. This could be due to development strategies derived from emotion and adapted to the limitations of life. In an ever-changing or uncertain environment, the fact that decisions are influenced by the prospect of immediate gains and losses could constitute a valuable survival strategy. Therefore, the myopic nature of such decisions may have survival-related significance. From the perspective of a limited life and bounded rationality (32), it would not be entirely irrational for decision-makers to develop myopic strategies based on gain–loss frequency.

The concepts of somatic markers and explicit knowledge, which were, respectively proposed by Damasio, Bechara et al. (10, 11), and Maia and McClelland (1), assumed that decision-makers are rational economic individuals (13, 14) who adopt foresighted strategies (1). The difference between gain–loss frequency and somatic markers and explicit knowledge is not unlike the difference between myopia and foresight, debates regarding which have been the focus of decision-making research discussions for the last 70 years (32, 33). A series of empirical results (34) gradually strengthened earlier hypotheses (32) regarding the role of bounded rationality in human decision-making. In an uncertain situation, a decision-maker may deal with the prospect of immediate gains and losses in a myopic but rational manner. Given the above-noted limitations of life, the implementation of myopic strategies could be a response to sudden changes in the environment, as well as to a rational rule of survival. We derived this alternative inference through our observation of participants' myopic decision strategies in the reanalysis in the present study of Maia and McClelland's original data. It is noteworthy that the clinical version of the IGT has been gradually utilized to assess, mostly based on the “foresighted” perspective, decision behavior for 13 types of psychiatric/neurological conditions. However, decision behavior cannot be said with certainty to be driven by “implicit emotion” or “explicit knowledge.” It should be noticed when experimenters or clinical psychiatrists interpret decision behavior by considering the “foresighted” perspective in the clinical version of the IGT (35, 36).

## Conclusions

Based on the basic assumption of long-term outcome, the research teams of Damasio and Maia and McClelland argued, respectively, that in the “late or early stage,” healthy decision-makers “have a hunch or know” the gain–loss structure of IGT. However, the present reanalysis points out that this argument might not be a critical issue. The key issue should be the study of why healthy decision-makers behave myopically in the IGT. Our reanalysis identified that the findings of studies (1, 10, 11), which maintain the same foresighted standpoint, were incongruent with those we obtained from the reanalyzed data of Maia and McClelland. Therefore, according to the present analysis, the Bechara et al. vs. Maia and McClelland debate, as featured in the April 2005 issue of *Trends in Cognitive Sciences* (13, 14), was unwarranted and should be reformulated. In short, we suggest that the issue of “What do participants in the IGT really know?” may still be controversial. However, we identified that participants behave based on gain–loss frequency.

## Author Contributions

Y-CC and C-HL initiated the present academic topic and literature review, defined the controversial issue, arranged the main structure, and refined the manuscript. Y-CC drafted the preliminary draft. W-KL and C-HL provided the data reanalysis, statistical testing, and parts of the data interpretation. J-TH, Y-CC, C-JL, and C-HL engaged in several rounds of critical discussion. C-HL and C-JL provided the final corrections and confirmations for all references. Y-CC, C-HL, W-KL, C-JL, and J-TH were responsible for the overall discussion and the final manuscript. All authors contributed to the article and approved the submitted version.

## Funding

The authors would like to thank the Ministry of Science and Technology (Taiwan) for its financial support under Contract No. NSC 102-2410-H031-014. C-HL's work was supported in part by the NSYSU-KMU JOINT RESEARCH PROJECT (#NSYSUKMU 109-I003; 110-I001).

## Conflict of Interest

The authors declare that the research was conducted in the absence of any commercial or financial relationships that could be construed as a potential conflict of interest.

## Publisher's Note

All claims expressed in this article are solely those of the authors and do not necessarily represent those of their affiliated organizations, or those of the publisher, the editors and the reviewers. Any product that may be evaluated in this article, or claim that may be made by its manufacturer, is not guaranteed or endorsed by the publisher.
