# Supplementary material for: Reanalyzing the Maia and McClelland (2004) Empirical Data: How Do Participants Really Behave in the Iowa Gambling Task?
Source: Front Psychiatry. 2022 Apr 8;13:788456. doi: 10.3389/fpsyt.2022.788456 (PMC9026173; doi:10.3389/fpsyt.2022.788456)
Supplement: Supplementary file 2 [file Data_Sheet_2.docx]

1. Firstly, we downloaded the open data of Steingroever et al. (2015) recollected from several IGT related studies as published as an open-source for further studies.

2. Secondly, we extracted a section of the dataset from Steingroever et al. (2015) database, which is provided by Maia and McClelland (2004). However, this dataset has no clear marks for identifying the replication and new questionnaire ((Bechara et al., 1997) vs. (Maia and McClelland, 2004)) groups. So, we further communicated with the authors (Profs. Maia and McClelland) to get the registration information and original data of the two groups.

3. The dataset extracted from Steingroever et al. (2015) was then co-registrated and rechecked with the original data of Maia and McClelland (2004).

4. According to Maia and McClelland's (2004) experimental condition marked, the dataset rearranged into the groups separately for easy comparison was shown in Supplementary Table 1.
