# Supplementary material for: Reanalyzing the Maia and McClelland (2004) Empirical Data: How Do Participants Really Behave in the Iowa Gambling Task?
Source: Front Psychiatry. 2022 Apr 8;13:788456. doi: 10.3389/fpsyt.2022.788456 (PMC9026173; doi:10.3389/fpsyt.2022.788456)
Supplement: Supplementary file 3 [file Data_Sheet_3.docx]

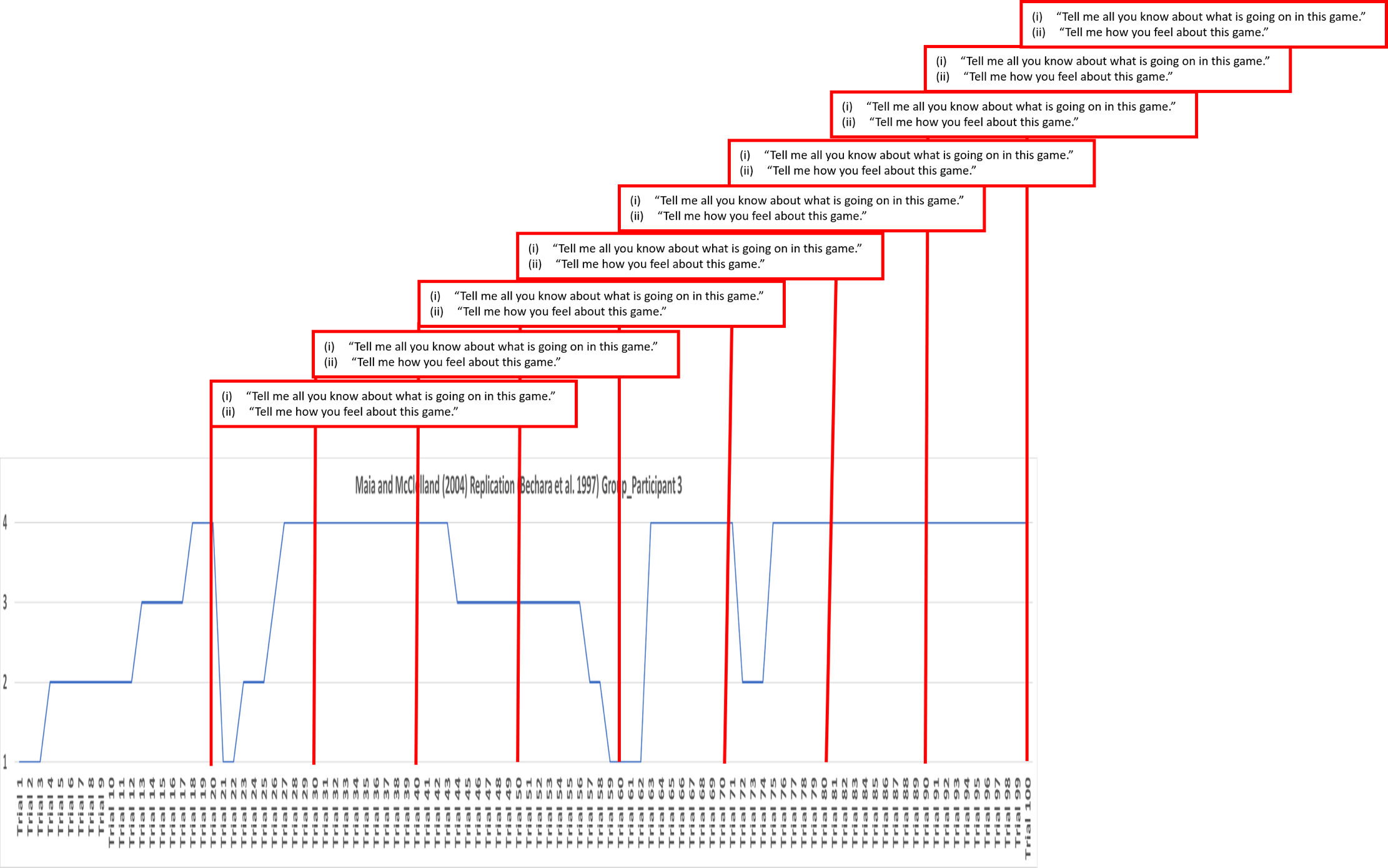


**Supplementary Figure 1 The questionnaire content and appearance time-point of Bechara et al. (1997) questionnaire.**

Here we depicted one participant’s IGT performance and the time-point of the questionnaire presented to the participant in the IGT. Notably, the Bechara et al. (1997) experimental procedure only presented two questions in each 10 card-turning since the first 20 trials. The two open questions were relatively simple than that of Maia and McClelland's (2004) version presented.


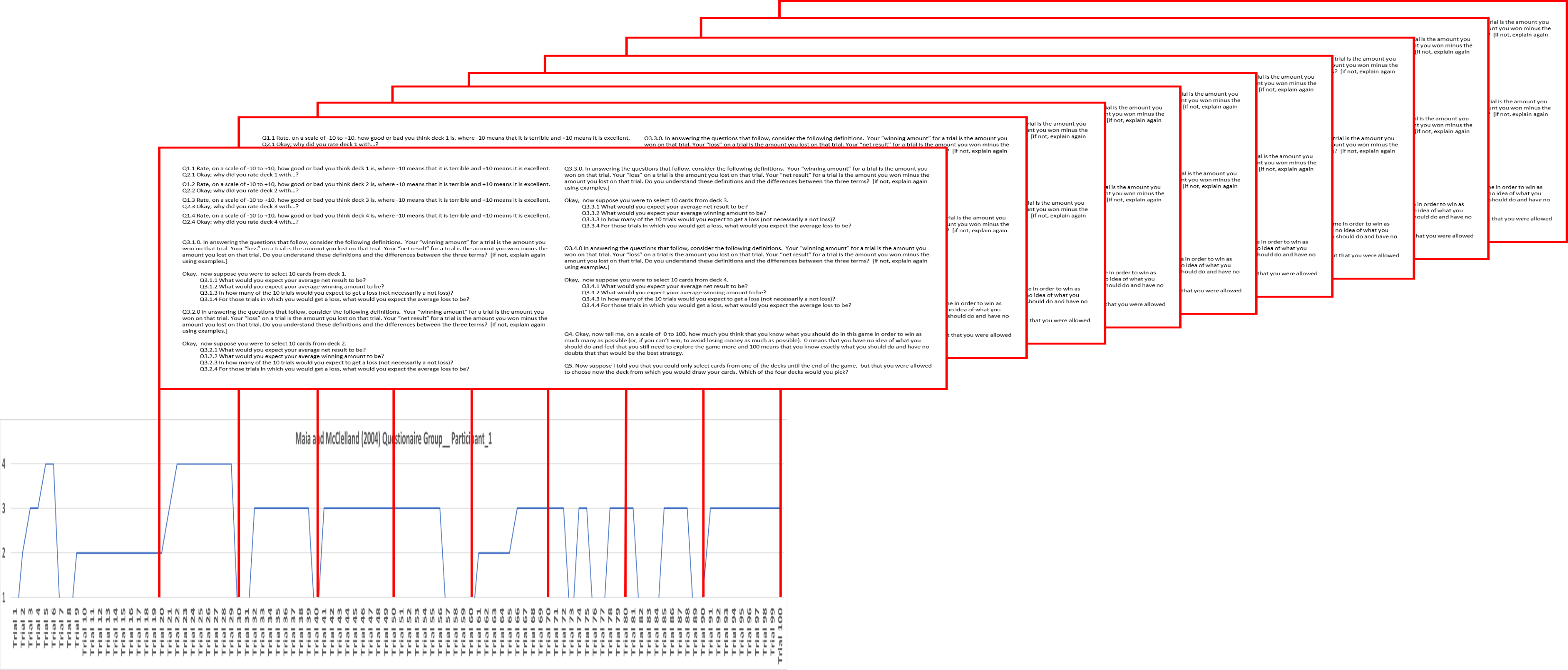


**Supplementary Figure 2 The questionnaire content and appearance time-point of Maia and McClelland's (2004) questionnaire.**

In this Figure 2, we showed one participant’s IGT performance and the time-point of the questionnaire presented to the participant in the IGT. Obviously, the content of Maia and McClelland's (2004) questionnaire is relatively detailed and lengthy than that of the Bechara et al. (1997) version presented. At each stop, participants not only have to answer the open questions but also answer some questions with the deck-by-deck procedure. Notably, the content and information of the two versions of the questionnaire presented/implicitly guided were totally different, so the future IGT studies should be carefully evaluated the debate of questionnaire content between Bechara et al. (1997) version vs. Maia and McClelland's (2004) version.
